# ICTV Virus Taxonomy Profile: *Solemoviridae* 2021

**DOI:** 10.1099/jgv.0.001707

**Published:** 2021-12-24

**Authors:** Merike Sõmera, Denis Fargette, Eugénie Hébrard, Cecilia Sarmiento, ICTV Report Consortium

**Affiliations:** ^1^​ Department of Chemistry and Biotechnology, Tallinn University of Technology, Tallinn 12618, Estonia; ^2^​ IRD, Cirad, Université Montpellier, IPME, Montpellier 34394, France

**Keywords:** ICTV Report, taxonomy, *Solemoviridae*

## Abstract

The family *Solemoviridae* includes viruses with icosahedral particles (26–34 nm in diameter) assembled on *T*=3 symmetry with a 4–6 kb positive-sense, monopartite, polycistronic RNA genome. Transmission of members of the genera *Sobemovirus* and *Polemovirus* occurs via mechanical wounding, vegetative propagation, insect vectors or abiotically through soil; members of the genera *Polerovirus* and *Enamovirus* are transmitted by specific aphids. Most solemoviruses have a narrow host range. This is a summary of the International Committee on Taxonomy of Viruses (ICTV) Report on the family *Solemoviridae*, which is available at ictv.global/report/solemoviridae.

## Abbreviations

CP, capsid protein; RdRP, RNA-directed RNA polymerase; sgRNA, subgenomic mRNA; VPg, virus protein genome-linked.

## Virion

**Fig. 1.**
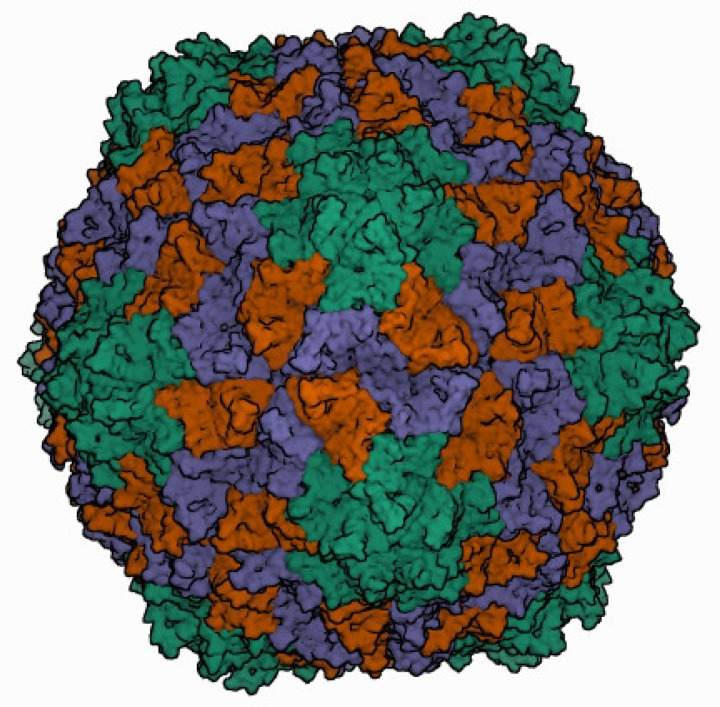
Three-dimensional cryo-electron reconstruction of the particle of rice yellow mottle virus shown at 2.8 Å resolution. The icosahedral asymmetric unit contains three subunits: A (in green), B (in brown), and C (in blue). Image from the RCSB PDB (rcsb.org) of PDB ID 1F2N [1].

Icosahedral virions of 26–34 nm in diameter are composed of 180 monomers of viral capsid protein (CP) on a *T*=3 lattice symmetry (Table 1). CP monomers can adopt three conformations designated as A, B and C (Fig. 1) [1]. Sobemovirus capsids are stabilized by divalent cations, pH-dependent protein–protein interactions and salt bridges between protein and viral RNA. Polerovirus and enamovirus particles lack proof of bound metal ions [2]. The N-terminal random domain of CP monomers is rich in basic amino acids and regulates the stability, curvature and assembly of virions. The C-terminal shell domain possesses a jellyroll β-sandwich topology, common in most non-enveloped icosahedral viruses. Virions of poleroviruses and enamoviruses incorporate a small proportion of CP with a 25–50 kDa C-terminal extension of a readthrough domain that is associated with aphid transmission and virus particle stability.

**Table 1.** Characteristics of members of the family *Solemoviridae*

Example:	southern bean mosaic virus (DQ875594), species *Southern bean mosaic virus*, genus *Sobemovirus*
Virion	Non-enveloped icosahedral particles with *T*=3 symmetry, 20–34 nm in diameter, composed of 180 molecules of capsid protein
Genome	4–6 kb positive-sense, non-segmented RNA, with 5′-terminal VPg; there is no poly(A) tail
Replication	Cytoplasmic
Translation	From genomic and subgenomic RNAs via leaky scanning, stop codon readthrough and −1 ribosomal frameshifting
Host range	Plants (monocotyledons and dicotyledons)
Taxonomy	Realm *Riboviria*, kingdom *Orthornavirae*, phylum *Pisuviricota*, class *Pisoniviricetes,* order *Sobelivirales*; four genera, including >55 species

## Genome

**Fig. 2.**
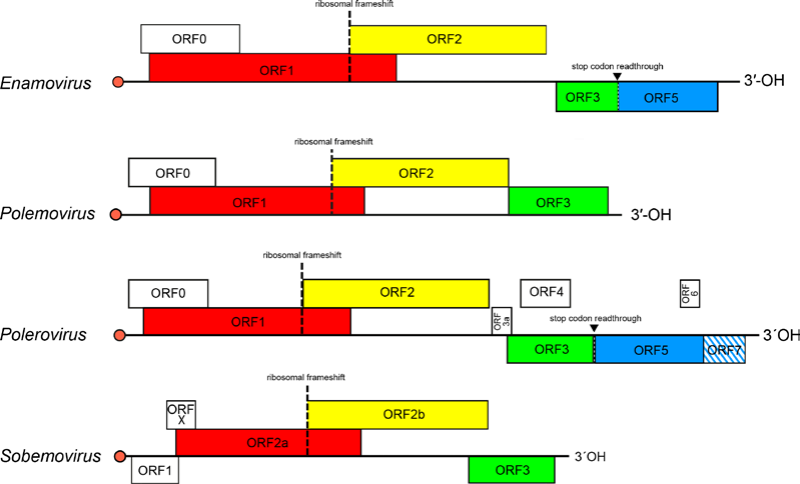
Genome organization of the members of the family *Solemoviridae*. Red bullets indicate 5′-VPg. ORF reading frames are shown by their position above the genome in the order −1, 0 and +1 and encode the polyprotein (red), RdRP (yellow), CP (green) and readthrough domain (blue). The hatched area indicates an overlap between ORFs.

The genome comprises a polycistronic, positive-sense RNA molecule of 4–6 kb without a 3′-poly(A) tail. The genome 5′-end has a covalently attached virus protein genome-linked (VPg). The genome contains 4–10 open reading frames (ORFs) (Fig. 2). The 5′-proximal ORF encodes a non-conserved RNA silencing suppressor protein, followed by a polyprotein that is expressed by ribosomal leaky scanning and cleaved autocatalytically to different functional subunits (membrane anchor, serine protease, VPg and C-terminal domains). Expression of the viral RNA-directed RNA polymerase (RdRP) as an alternative C-terminal domain of the polyprotein is regulated by a −1 programmed ribosomal frameshift. A 3′-proximal ORF of solemoviruses encodes CP, expressed from a subgenomic mRNA (sgRNA). For polero- and enamoviruses, suppression of an amber stop codon of the CP gene leads to translation of readthrough domain protein from ORF5. Other ORFs are genus-specific.

## Replication

Replication occurs after synthesis of RdRP from genomic RNA. A conserved 5′-end sequence, 5′-ACAA(AA)−3′, and a stable 3′-end stem–loop are essential for template recognition. The VPg of cereal yellow dwarf virus RPV acts as a primer for full-length negative-sense strand RNA synthesis [3], whereas primer-independent replication has been demonstrated for Sesbania mosaic virus [4]. Sobemoviruses, polemoviruses and enamoviruses transcribe one sgRNA; poleroviruses generate up to three sgRNAs.

## Pathogenicity

Infections can be symptomless or cause severe diseases. The family includes important plant pathogens with high economic impact, of which rice yellow mottle virus, potato leafroll virus, sugarcane yellow leaf virus and beet-infecting poleroviruses are the most devastating. Virulent infections induce chlorosis, phloem necrosis, stunting and sometimes sterility. Sobemoviruses infect different tissue types, whereas poleroviruses and enamoviruses are phloem-specific. Whereas sobemoviruses are mainly transmitted via mechanical wounds [5], transmission of poleroviruses and enamoviruses is by aphids in a persistent, circulative and non-propagative mode [6].

## Taxonomy

Current taxonomy: ictv.global/taxonomy. The genera *Sobemovirus* and *Polerovirus* each include 20 or more species and the genus *Enamovirus* includes 5 species. The genus *Polemovirus* includes the species *Poinsettia latent virus*.

## Resources

Full ICTV Report on the family *Solemoviridae*: ictv.global/report/solemoviridae.
